# Adaptive Immune Response to Model Antigens Is Impaired in Murine Leukocyte-Adhesion Deficiency-1 Revealing Elevated Activation Thresholds *In Vivo*

**DOI:** 10.1155/2012/450738

**Published:** 2012-03-04

**Authors:** Thorsten Peters, Wilhelm Bloch, Oliver Pabst, Claudia Wickenhauser, Claudia Uthoff-Hachenberg, Susanne V. Schmidt, Georg Varga, Stephan Grabbe, Daniel Kess, Tsvetelina Oreshkova, Anca Sindrilaru, Klaus Addicks, Reinhold Förster, Werner Müller, Karin Scharffetter-Kochanek

**Affiliations:** ^1^Department of Dermatology and Allergic Diseases, University of Ulm, Maienweg 12, 89081 Ulm, Germany; ^2^Department of Molecular and Cellular Sports Medicine, German Sports University, Am Sportpark Müngersdorf 6, 50933 Cologne, Germany; ^3^Institute of Immunology, Hannover Medical School, Carl-Neuberg-Straß 1, 30625 Hannover, Germany; ^4^Institute of Pathology, University of Leipzig, Liebigstraße, 04103 Leipzig, Germany; ^5^Mouse Genetics and Inflammation Laboratory, Institute for Genetics, University of Cologne, Zülpicher Straß 47a, 50674 Cologne, Germany; ^6^LIMES (Life and Medical Sciences) Institute, University of Bonn, Carl-Troll-Straß 31, 53115 Bonn, Germany; ^7^Institute of Immunology, University of Muenster, Röntgenstraße 21, 48149 Muenster, Germany; ^8^Department of Dermatology, University of Mainz, Langenbeckstr. 1, 55131 Mainz, Germany; ^9^Department of Molecular Immunology, Institute of Biology and Immunology of Reproduction, Bulgarian Academy of Sciences, 73 Tzarigradsko shose, 1113 Sofia, Bulgaria; ^10^Department of Anatomy I, University of Cologne, Joseph-Stelzmann Straß 9, 50931 Cologne, Germany; ^11^Faculty of Life Science, University of Manchester, Oxford Road, Manchester M13 9PT, UK

## Abstract

Absence of **β**_2_ integrins (CD11/CD18) leads to leukocyte-adhesion deficiency-1 (LAD1), a rare primary immunodeficiency syndrome. Although extensive *in vitro* work has established an essential function of **β**_2_ integrins in adhesive and signaling properties for cells of the innate and adaptive immune system, their respective participation in an altered adaptive immunity in LAD1 patients are complex and only partly understood *in vivo*. Therefore, we investigated adaptive immune responses towards different T-dependent antigens in a murine LAD1 model of **β**_2_ integrin-deficiency (CD18^−/−^). CD18^−/−^ mice generated only weak IgG responses after immunization with tetanus toxoid (TT). In contrast, robust hapten- and protein-specific immune responses were observed after immunization with highly haptenated antigens such as (4-hydroxy-3-nitrophenyl)_21_ acetyl chicken **γ** globulin (NP_21_-CG), even though regularly structured germinal centers with specificity for the defined antigens/haptens in CD18^−/−^ mice remained absent. However, a decrease in the hapten/protein ratio lowered the efficacy of immune responses in CD18^−/−^ mice, whereas a mere reduction of the antigen dose was less crucial. Importantly, haptenation of TT with NP (NP-TT) efficiently restored a robust IgG response also to TT. Our findings may stimulate further studies on a modification of vaccination strategies using highly haptenated antigens in individuals suffering from LAD1.

## 1. Introduction

Adaptive immune responses require the integration of complex antigen receptor and costimulatory signals on T cells as well as B cells to override activation thresholds that lead to lymphocyte differentiation and eventually, to antibody production. The *β*_2_ integrin LFA-1 (CD11a/CD18) is particularly prominent in promoting the interaction between APC and naïve T cells [1] as well as between T cells and B cells [2, 3]. In this, LFA-1 signaling is an important determinant of phenotypic outcome in naïve T-cell and B-cell maturation and effector function [4–6].

A prerequisite for T-cell activation is a stable T-cell stimulation which is essential to be sustained up to several hours [4] to achieve commitment to cytokine synthesis (IL-2, IFN-*γ*) [7–9]. It has been reported that the duration of stimulation directly contributes to the commitment of CD4^+^ T cells to division thus correlating with the development of effector functions and the extent of memory generation [10]. Furthermore, it has been assumed that the adhesive interaction mediated by LFA-1 increases the avidity of T cell : APC interaction allowing T cells to sample large numbers of specific peptides presented on MHC class II on the surface of APC which then leads to a more efficient TCR engagement. Increased adhesion can thus lower the effective dose of antigen required to reach a minimal threshold number of activated TCR complexes [1, 5, 11, 12]. Furthermore, LFA-1 facilitates the clustering of surface molecules such as the TCR, CD28, and CD40 in the immunological synapse between T cells and APC (quantitative signal enhancement) [13, 14]. In addition to its above-described adhesive functions, LFA-1 may also provide unique signals that are primarily independent of TCR signaling (qualitative signal modulation) [5, 8]. Model systems used to study T-cell activation have shown that even a mere increase in antigen “quantity” at a large scale could not initiate naïve CD4^+^ T-cell proliferation or cytokine synthesis in the absence of ICAM/LFA-1 interaction [15]. Collectively, it is well established that LFA-1 stimulation increases the number of activated T cells favoring Th1 differentiation, distinctly enhances activation velocity in particular by supporting early IL-2 production, leading to entry of naïve cells into the cell cycle and reduces activation thresholds of T cells. Hence, LFA-1/ICAM signaling significantly supports T-cell activation and polarization towards the Th1 phenotype [5, 7]. In the absence of functional LFA-1, Th1 differentiation is severely impaired, whereas Th2 response is strongly enhanced, both *in vitro* and *in vivo* [9, 16, 17].

Interestingly, the relevance of LFA-1/ICAM for T : B immunological synapses has also been documented for membrane-mediated B-cell activation by Th cells [2]. Signaling from antigen-specific T cells to B cells to induce activation required LFA-1/ICAM-1 ligation and is based on tight physical contact of T : B in an immune synapse [18]. In this context, Carrasco and coworkers showed that inclusion of ICAM-1 in the immunological synapse decreases the B-cell avidity threshold by at least 10-fold [2]. At low antigen densities, LFA-1 can help B-cells adhering, forming a synapse, and becoming activated. Thus, in analogy to the T cell : APC interaction, synergy of BCR crosslinking and ICAM-1-mediated signals can reduce threshold barriers for B-cell activation. Vice versa, effective B : T cell synapses are of even greater importance for T-cell activation by antigen-presenting B cells (B-APC). Engagement of the BCR by polyvalent antigen can rapidly elicit expression of B7-2 (CD86) on B cells resulting in a robust costimulatory signal that is sufficient even to drive naïve Th cell responses [19, 20].

Although detailed studies on the adhesive and differentiation-inducing functions of LFA-1-mediated binding for APC : T cell and T : B cell contacts are available, it still remains incompletely understood how the observed *in vitro* functions combine and contribute to the clinical picture of immunodeficiency in individuals lacking *β*_2_ integrins *in vivo*. Absence of *β*_2_ integrins (CD11/CD18) in humans leads to leukocyte-adhesion deficiency-1 (LAD1), a severe primary immunodeficiency syndrome. Expression of less than 1% of CD18 causes a severe form of the disease with recurrent life-threatening bacterial or fungal infections resulting in death of patients early in childhood [21, 22]. Impaired adaptive immune responses to common vaccination protocols have been observed as one important feature of the disease [23, 24]. We have previously reported on a murine model for LAD1 carrying a CD18 null mutation that shares all major features with the human syndrome [25–29]. Using this murine model, we herein dissect the role of *β*_2_ integrins in functional and structural components of the adaptive immune response *in vivo*.

Our results demonstrate that in absence of *β*_2_ integrins, mice display a severely impaired adaptive immune response* in vivo*. A marked elevation of activation thresholds excluded the commonly potent antigen TT as an immunogen, whereas haptenation of carrier proteins could override the activation threshold and elicited robust adaptive immune responses. These findings indicate that modifying vaccination approaches towards the use of highly haptenated antigens may be more successful in LAD1 patients.

## 2. Materials and Methods

### 2.1. Mice

All mice were maintained on a mixed 129Sv × C57BL/6 background. CD18^−/−^ homozygotes [25] and CD18^+/+^ WT controls were derived from heterozygote crosses. Immunization trials were performed under specific pathogen-free (SPF) conditions using mice at an age of 8–12 weeks. All experiments were done in compliance with the German Law for Welfare of Laboratory Animals.

### 2.2. Immunization of CD18^−/−^ Mice

For immunization of mice, protein antigens conjugated with a hapten at different haptenation ratios were used. These hapten-coupled proteins were prepared according to a previously established protocol [30, 31]. In brief, per animal, a solution of 200 *μ*L PBS containing 10 or 100 *μ*g of (4-hydroxy-3-nitrophenyl) acetyl (NP; Bioresearch Technologies, Inc., Novato, CA) coupled to chicken *γ* globulin (CG; Calbiochem, Schwalbach, Germany) with a ratio of 21 or 4 NP molecules per molecule CG was precipitated by adding 200 *μ*L 10% KAl(SO_4_)_2_ (alum; Merck Chemicals, Darmstadt, Germany) and was then titrated using 5 N NaOH. Or else, uncoupled CG was precipitated. The alum precipitates were injected intraperitoneally into mice. All animals were reinjected with 10 or 100 *μ*g of the soluble, unprecipitated NP-CG or CG at the indicated time points.

Immunization with tetanus toxoid (TT) was performed in a similar fashion. “Tetanus-Impfstoff Mérieux” vaccination suspension was purchased from Aventis Pasteur MSD (Lyon, France). Eight- to tewlve-week-old mice were injected with a dose of either 2.0 Lf (flocculation units) or 0.2 Lf of alum-precipitated TT. In a further trial, mice were immunized with 2.0 Lf of alum-precipitated TT haptenated with NP molecules at an unknown ratio, according to a previously published protocol for the hapten conjugation of protein carrier molecules [30, 31]. All mice were reinjected with the same dose of TT or NP-TT, respectively, at indicated time points of the trials.

### 2.3. Measurement of NP-Specific Ig

Immune responses were estimated by ELISA detection of NP hapten-specific IgM, IgG_1_, *κ*, and *λ* light chain Ab in the sera of mice immunized with NP-CG as described elsewhere [30, 32]. Ninety-six-well plates (Greiner Bio-One, Frickenhausen, Germany) were coated with 10 *μ*g/mL NP_14_-BSA in PBS at 4°C overnight and were then blocked with 0.5% BSA in PBS. Serially diluted sera obtained at the indicated time points after immunization were added and incubated at 4°C overnight. On each plate, equally diluted anti-NP mAb standards with corresponding isotypes (prepared at the Institute of Genetics, University of Cologne according to previously published protocols [30, 31]) were included to obtain appropriate standard curves. After intermittent washing steps with H_2_O, biotinylated detection antibodies (goat anti-mouse IgM, IgG, *κ* and *λ*; Southern Biotechnology Associates Inc., Birmingham, AL) at a dilution of 1 : 1000 and alkaline-phosphatase-(ALP-) conjugated streptavidin (1 : 3000; Roche, Mannheim, Germany) were added. ALP activity was visualized using ALP substrate solution (0.4 mg/mL; Roche) and subsequently, OD was measured at 405 nm versus 570 nm. The concentrations were determined by comparing to standard curves created from above mentioned anti-NP standards.

### 2.4. Affinity Maturation of NP-Specific Antibodies

Assessment of affinity maturation of NP-specific antibodies was carried out by ELISA using two different coupling ratios of NP-BSA as described previously [32, 33]. Briefly, 96-well plates (Greiner) were coated with 10 *μ*g/mL NP_4_-BSA or NP_14_-BSA in PBS at 4°C overnight. After blocking with 0.5% BSA in PBS, sera obtained at the indicated time points after immunizations were serially diluted and plated out. On each plate, anti-NP-specific mAb standards of the same isotype but with different affinity constants (*Ka*) (prepared at the Institute of Genetics, University of Cologne according to previously published protocols [30, 31]) were added to obtain standard curves for affinity assessment. The final steps of the ELISA were then performed as described above using biotinylated goat anti-mouse *λ* light chain and IgG_1_ (data not shown) detection Ab. To estimate the affinity of NP-binding antibody in the sera, ratios of NP_4_-binding antibody to NP_14_-binding antibody were calculated.

### 2.5. Measurement of Protein-Carrier-Specific IgG

For assessment of anti-TT- or anti-CG-specific IgG Ab, sera obtained by bleeding from tail veins were analyzed by ELISA. Briefly, for anti-TT detection, human Tetanus IgG ELISA kits were purchased from IBL (Hamburg, Germany) and ELISA performed according to a slightly modified protocol, as distributed by the manufacturer. Sera were initially diluted 1 : 10 in assay diluent and subsequently plated out in 1 : 5 or 1 : 6 dilution steps using assay diluent. For detection of murine anti-TT IgG Ab, a horseradish peroxidase-conjugated rat anti-mouse IgG mAb (X56; Pharmingen, BD, Heidelberg, Germany) was used at a dilution of 1 : 1000. Tetramethylbenzidine (TMB, IBL) served as a substrate for the color reaction. Plates were read at 450 nm within 60 minutes after addition of 1 M H_2_SO_4_. Anti-TT IgG titers were calculated from the last dilution step where the OD was still above the background level. Assays for measurement of anti-CG IgG were performed accordingly, except with the modification that, initially, 96-well plates (Greiner) were coated with 10 *μ*g/mL soluble CG in PBS and were blocked with 0.5% BSA. Subsequently, all further procedures were carried out as described above.

### 2.6. Antibodies and Fluorochrome-Coupled Proteins

GL7-FITC (Ly77), CD18-PE (C71/16) mAbs were purchased from Pharmingen, CD19-PE (6D5) was from SBA, and IgD (HB250) and IgM (HB88) were obtained as described earlier [34]. Peanut agglutinin (PNA)-FITC was purchased from Sigma (Taufkirchen, Germany) and DAPI from Roche (Grenzach-Wyhlen, Germany).

For detection of antigen-specific cells, NP_4_-CG and CG were labeled with Cychrome 5 (Cy5), and conjugates purified using NAP columns, as recommended by the manufacturer (Amersham/Pharmacia, Freiburg, Germany). NP_4_-CG and CG were used instead of NP_21_-CG for fluorochrome coupling to allow sufficient binding of Cy5 to free sites of the CG.

### 2.7. Histology

Spleens were removed at the indicated time points after immunization and were embedded in Tissue-Tek O.C.T. compound (Fisher Scientific, Bridgewater, NJ) for cryosections. Immunohistologic analysis of adult lymphoid tissues was done as described earlier [34] using a motorized Axiovert M200 microscope (Carl Zeiss, Germany). Frozen sections of 6–10 *μ*m thickness were mounted on slides and fixed in cold acetone. Cryosections were blocked with rat serum and stained with mAb and lectins against the indicated markers. Overviews of spleen sections shown in Figure 3(a) were achieved using automated image assembly applying the KS300 MosaiX software (Carl Zeiss, Oberkochen, Germany).

### 2.8. FACS Analysis

Cells were obtained from spleens, or from BM flushed out of femurs of mice. Red blood cells were removed using an osmotic lysis buffer (0.15 M NH_4_Cl, 1.0 M KHCO_3_, 0.1 M Na_2_EDTA, pH 7.2). The remaining leukocyte fraction was adjusted to 1 × 10^6^ cells per 50 *μ*L and unspecific binding was blocked with 2% rat serum. Subsequently, 50 *μ*L of the cell suspension were stained with ≤1 *μ*L of the fluorochrome-conjugated mAbs (at a stock concentration of 0.5–1 mg/mL, dependent on the respective mAb-fluorochrome conjugate) for 30 min at 4°C. Stained cells were analyzed using a FACSCalibur (BD, Heidelberg, Germany).

### 2.9. Statistics

For statistical evaluation of the differences in serum Ig levels, Mann-Whitney *U* test was used. Differences were considered statistically significant when *P* < 0.05.

## 3. Results

### 3.1. Impaired Humoral Immune Response in CD18^−/−^ Mice upon Immunization with Tetanus Toxoid

**Figure 1:**
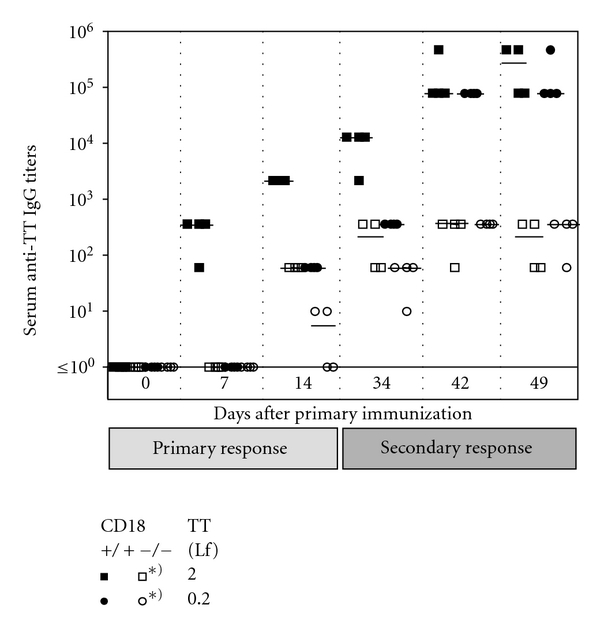
Defective humoral immune response upon TT in CD18^−/−^ mice. Eight- to twelve-week-old CD18^−/−^ (open symbols) and WT (filled symbols) mice were immunized intraperitoneally with 2.0 (squares) or 0.2 Lf (circles) of tetanus toxoid (TT)/alum. Animals were reimmunized with the same dose of the antigen at day 34. For assessment of the primary immune response, sera were collected at days 0, 7, and 14, for secondary immune response at days 34, 42, and 49. Subsequently, sera were diluted 1 : 10, and plated out on TT-coated plates in 1 : 6 dilution steps. Serum titers of anti-TT specific IgG_1_ were determined from the last dilution step where the optical density was still above the background level of the assay. Bars represent the median of each group. *Indicates a *P* < 0.05 for the marked cohorts at all times points shown, from day 14 on.

LAD1 patients suffer from a severe immunodeficiency due to an absence of functional CD18 heterodimers. Patients [23, 24] as well as cattle [35] deficient in CD18 have been described to respond poorly to T-dependent antigens or vaccines such as bacteriophage *ϕ*X174 or tetanus toxoid (TT). Since TT is a well-characterized and commonly potent immunogen that has been frequently employed to detect T-dependent immunodeficiency by others before, we determined anti-TT IgG titers in a TT vaccination trial in the murine LAD1 model. CD18^−/−^ and WT mice were immunized with either 2.0 or 0.2 flocculation units (Lf) of TT/alum. For assessment of memory B-cell function and amplification of specific Ig production during secondary immune response, animals were boost-immunized with the same doses at day 34. Serum levels of anti-TT IgG were detected by ELISA at different time points throughout the trial. At all time points analyzed, anti-TT IgG titers were significantly lower in CD18^−/−^ mice than in WT controls independent of the TT dose injected (*P* < 0.05) (Figure 1). After secondary immunization, anti-TT IgG titers of CD18^−/−^ mice were about three logs below WT control titers. Whereas in WT mice a strong amplification of the immune response occurred, CD18^−/−^ mice were not able to amplify their anti-TT IgG production any further after reimmunization with the antigen. However, TT-specific IgG titers were measurable also in CD18^−/−^ mice, confirming that class switch was not impaired.

### 3.2. Robust T-Dependent Humoral Immune Response in CD18^−/−^ Mice upon Immunization with NP-CG

**Figure 2:**
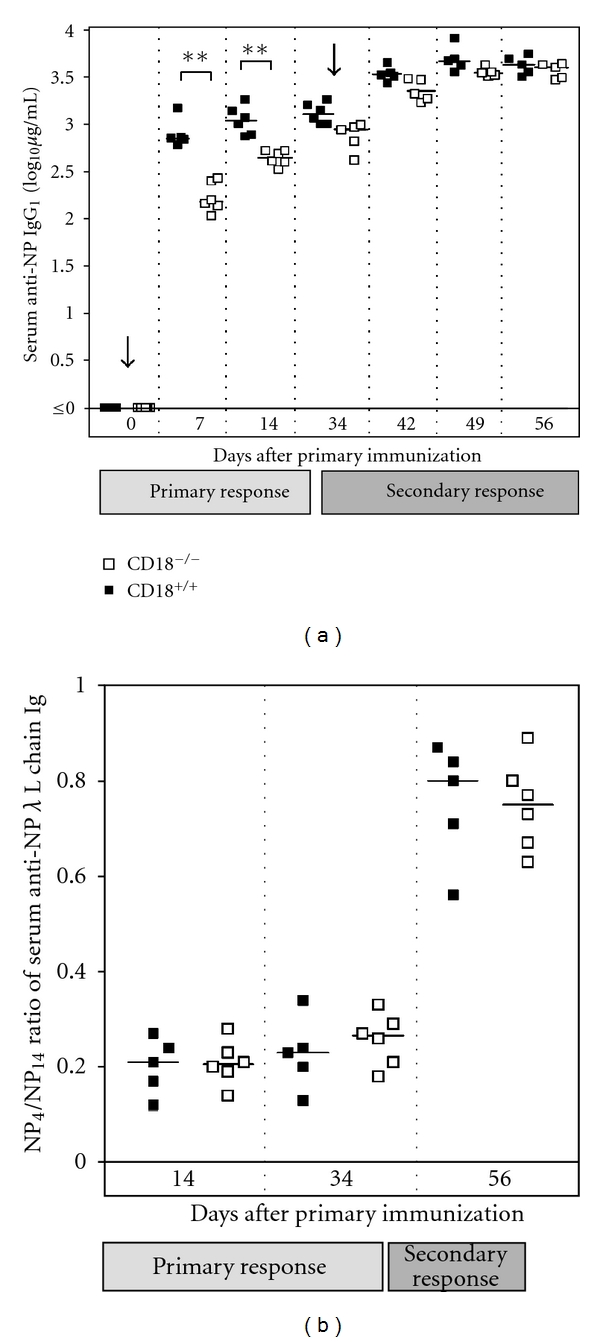
Functional humoral immune response upon NP-CG in CD18^−/−^ mice. Eight- to twelve-week-old CD18^−/−^ and WT mice were immunized intraperitoneally with 100 *μ*g of alum-precipitated NP-CG. Animals were reinjected with 100 *μ*g of soluble NP-CG at day 34. Sera were collected at days 0, 7, and 14 during primary immune response, and at days 34, 42, 49, and 56 during secondary immune response. (a) Serum levels of anti-NP-specific IgG_1_ were subsequently detected by ELISA on NP_4_-coated ELISA plates and calculated by comparison to an IgG_1_ standard. (b) Anti-NP-specific Ig carrying *λ* or *κ* L chains were differentially detected on high-density (NP_14_-BSA) and low-density (NP_4_-BSA) hapten-coated ELISA plates in sera obtained at days 14, 34, and 56. Affinity maturation of NP-specific antibodies was estimated as ratio of NP_4_- to NP_14_-binding antibodies for each of the three time-points. Bars represent the median of each group. **P < 0.005.

To address the question, whether defective adaptive immunity in CD18^−/−^ mice relied on particular TT-specific properties, CD18^−/−^ and WT mice were immunized with the alum-precipitated antigen NP_21_-CG at a dose as high as 100 *μ*g per mouse, in an analogous immunization trial. For measurement of memory B-cell function and amplification of specific Ig production during secondary immune response, animals were boost-immunized with 100 *μ*g of soluble NP_21_-CG at day 34. Serum levels of anti-NP-specific Ig were detected and further differentiated into subclasses by ELISA. Surprisingly, a slightly lower production of anti-NP IgG_1_ was detectable only during the primary immune response in CD18^−/−^ mice (Figure 2(a)). At day 7, titers of CD18^−/−^ mice were about 4.5-fold reduced when compared to WT controls (*P* < 0.005), whereas at day 14, this difference had decreased to 2.5-fold (*P* < 0.005). No significant differences in NP-specific Ig titers of CD18^−/−^ and control mice occurred after rechallenge with the soluble antigen, from day 42 onwards (*P* > 0.05). These results demonstrate a slight shift in the kinetics of the primary immune response in CD18^−/−^ mutants, with an initial decrease in hapten-specific IgG_1_ production, whereas primary NP-specific IgM were not reduced in CD18^−/−^ mice (data not shown). However, overall hapten-specific IgG peak titers mounted by CD18^−/−^ mice after immunization were in the same range as in WT controls showing that class switch as such was not impaired. After boosting, amplification of the immune response was as high in CD18^−/−^ as in WT mice reflecting a normal memory B-cell generation and function. Besides, antibody composition of either *κ* or *λ* light chains was comparable to those of WT controls and revealed a marked prevalence of *λ* light chains during the primary IgG response to NP, a typical feature of the C57BL/6 mouse strain (data not shown) [30].

To determine affinity maturation of NP-specific antibodies, sera collected during the immunization trial were analyzed for their contents of low and high affinity antibodies for the hapten NP. Our data clearly demonstrate that affinity maturation occurred to the same extent in CD18^−/−^ as in WT mice, both showing a sharp increase in anti-NP affinity after repeated antigen challenge (*P* < 0.05) (Figure 2(b)).

### 3.3. No Formation of Antigen-Specific GC after Immunization with NP_21_-CG

**Figure 3:**
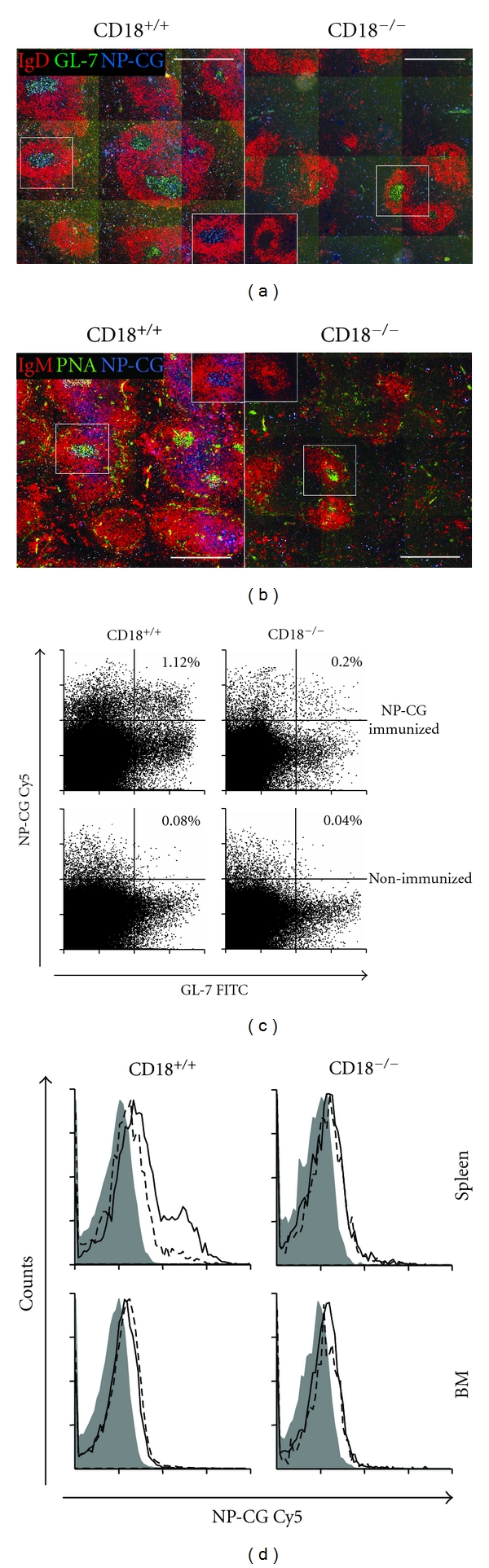
No induction of GC with NP_21_-CG specificity after immunization in CD18^−/−^ mice. Two weeks after immunization, spleens and BM of WT and CD18^−/−^ mice were subjected to immunofluorescence microscopy (spleens; (a), (b)) or flow cytometry (BM, spleens; (c), (d)). B-cell follicles identify as IgD^+^ (b) or IgM^+^ (a) (both in red), whereas GC locate within the follicles and stain IgD^−^ GL-7^+^ (a) or IgM^−^ PNA^+^ (b) (GL-7 and PNA both in green). Specificity for NP-CG was assessed by staining with NP-CG-Cy5 (blue). (a) and (b) show representative sections of spleens solely from immunized mice. Scale bars, 500 *μ*m. Spleen ((c); (d), upper histograms) or BM ((d), lower histograms) cells were analyzed by FACS. (c) displays representative dot plots of splenic cells gated for CD19^+^ IgM^low^. B cells with a GC phenotype additionally stain positive for GL-7 and are situated in the right quadrants of each plot. GC B cells with specificity for NP-CG are located in the upper right quadrants. Percentages for size-gated cells are indicated for immunized (upper dot plots) and mock-immunized (lower dot plots) mice. (d) shows representative histogram plots of cells gated for CD19^+^ IgM^low^ GL-7^+^, representing GC B cells. The continuous lines indicate samples obtained from mice immunized with NP_21_-CG; dashed lines: mock-immunized mice; grey areas: irrelevant control conjugates.

CD18^−/−^ mice have a severely disturbed architecture of secondary lymphoid organs such as the spleen and the lymph nodes [25, 28, 36]. Since adaptive immunity in CD18^−/−^ mice was nevertheless functional upon immunization with NP_21_-CG, we set out to detect germinal centers with specificity for the injected antigen NP_21_-CG. To exclude artefacts skewing histological analysis of CD18^−/−^ mice, mice were used for histology at an age of 8–12 weeks when lymphoid architecture had not yet succumbed to secondary lymphoid and myeloid hyperplasia. As described above, CD18^−/−^ and control mice were injected with 100 *μ*g NP_21_-CG/alum. Secondary lymphoid tissues were removed at day 14, when GC formation in mice is at its maximum (Figure 3). Cryosections of WT spleens showed typical germinal centers (GL-7^+^PNA^+^, IgD^−^IgM^−^) with numerous GC that stained positive for the antigen NP-CG coupled with Cy5 (NP-CG-Cy5) (Figures 3(a) and 3(b)). In contrast to WT, CD18^−/−^ mice had considerably fewer GC, of a smaller size and altered structure. In addition, none of the GC-like structures but only some disseminated cells stained for the antigen NP_21_-CG in CD18^−/−^ mutants. Importantly, these cells were situated extrafollicularly and were not organized in clusters as are GC.

Since no classical NP-CG-Cy5^+^ GC structures could be detected in immunized CD18^−/−^ mice by histology, whereas IgG with high affinity for NP_21_-CG was abundant in the sera, we analyzed lymphoid tissues for NP-CG-Cy5^+^ cells with a GC-like phenotype (CD19^+^GL7^+^ or CD19^+^PNA^+^) [32, 37] by flow cytometry (Figures 3(c) and 3(d)). As observed by immunofluorescent microscopy, NP_21_-CG immunized WT revealed a prominent CD19^+^ B cell population that stained for the GC marker GL-7, or PNA (data not shown), and NP-CG-Cy5. In contrast, in CD18^−/−^ mice, CD19^+^ B-cells staining double positive for GL-7 and NP-CG-Cy5 were 5 times less frequent compared to WT mice (Figure 3(c)). Hence, GC-like cells specific for NP-CG were present, but without a distinct structural correlative in histology.

In case of altered lymphocyte trafficking and disrupted secondary lymphoid tissue, BM can function as site of primary immune response [38]. B cells, which have acquired a typical PNA^+^ GC phenotype, have been described to seed to the BM, where they further differentiate into antibody-forming cells (AFCs) [39]. Thus, BM may, to some extent, serve as a refuge for late B-cell development and maturation. To trace B cells showing a GC phenotype in BM [33] of CD18^−/−^ mice, mononuclear cells were isolated from BM and stained for GC markers. Only very few CD19^+^IgM^low^GL-7^+^ cells could be detected using flow cytometry. These cells did not differ in fluorescence intensities for NP-CG-Cy5 in NP_21_-CG immunized and nonimmunized cohorts, neither in CD18^−/−^ nor in WT mice (Figure 3(d)). Thus, in CD18^−/−^ mice no evidence for a compensatory function of the BM in hosting GC-like cells was detected. Our data reveal a ubiquitous deficiency for specific GC formation upon immunization with NP_21_-CG in all lymphoid tissues of CD18^−/−^ mice subjected to analysis, postulating a salvage mechanism or alternative pathway in generating high-affinity AFC that yet remains unclear.

### 3.4. Efficacy of the Immune Response Directly Correlates to the Hapten/Protein Ratio of Antigens in CD18^−/−^ Mice

**Figure 4:**
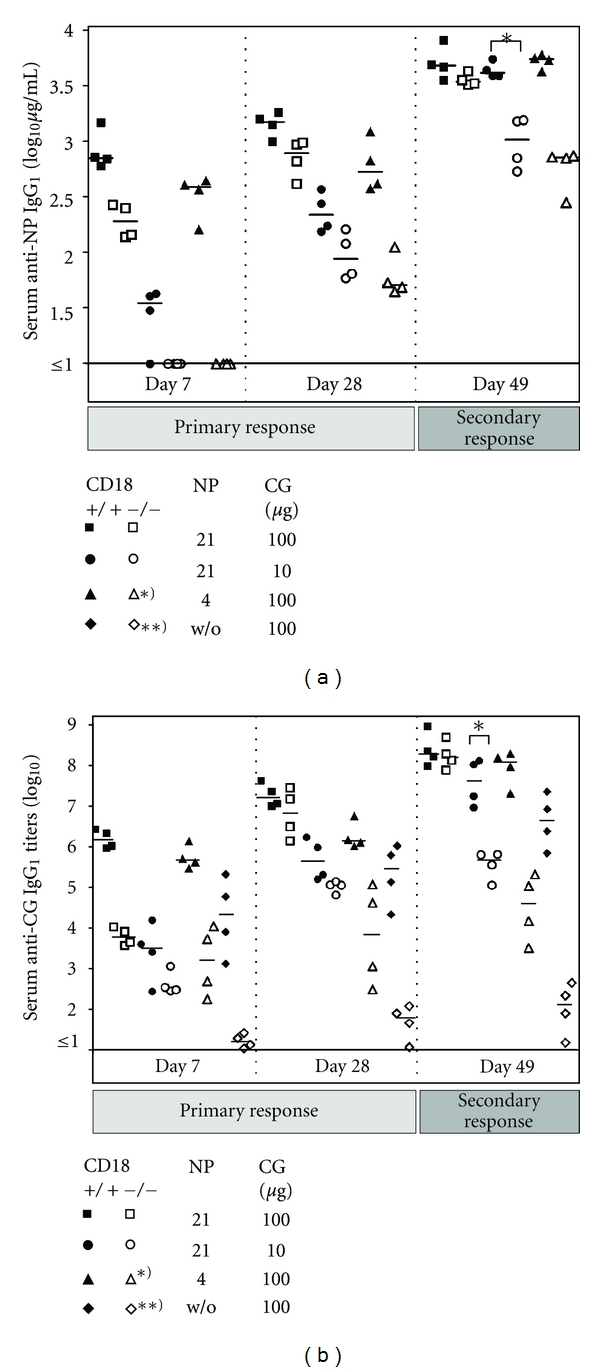
High NP/CG ratios are critical for elicitation of hapten- and protein-specific IgG titers in CD18^−/−^ mice. Eight- to twelve-week-old CD18^−/−^ (open symbols) and WT (filled symbols) mice were immunized intraperitoneally with 100 *μ*g of NP_21_-CG/aluM (squares), 10 *μ*g NP_21_-CG/alum (circles), 100 *μ*g NP_4_-CG/alum (triangles), or 100 *μ*g CG/alum (diamonds). (a) Serum levels of anti-NP specific IgG_1_ were subsequently detected by ELISA on NP_4_-BSA coated ELISA plates and calculated in *μ*g/mL by comparison to IgG_1_ standards, as described above. (b) For detection of CG-specific IgG_1_, sera obtained by bleeding from tail veins were diluted 1 : 10, and then plated out on CG-coated plates in 1 : 5 dilution steps. Serum titers of anti-CG-specific IgG_1_ were determined from the last dilution step where the optical density was still above the background level of the assay. For assessment of the primary immune response, results from sera collected at days 7 and 28 are displayed. Besides, measurements for day 49 are shown, and depict IgG_1_ titers representative also for further time points assessed during secondary immune responses. Bars represent the median of each group. *P < 0.05; **P < 0.005. Asterisks used in the key box indicate significant differences for the marked cohorts at all times points shown.

To better understand potential reasons for the in part contradictory results obtained by immunization with NP_21_-CG and TT, we modified our immunization protocols with regard to antigen quantity and quality. Given the high dose of 100 *μ*g NP_21_-CG administered in the initial trial, we repeated the experiment with a low dose injecting 10 *μ*g NP_21_-CG/alum for the induction of primary immune responses, and 10 *μ*g of soluble NP_21_-CG/PBS for boosting. Furthermore, to test whether the degree of haptenation may be crucial for the induction of a full immune response in CD18^−/−^ mice, we have used a reduced NP/CG ratio of 4/1, or nonhaptenated CG without NP. This time, besides anti-NP specific IgG, also anti-CG-specific IgG were determined from the sera to rule out that in CD18^−/−^ mice, hapten-specific responses may be functional but carrier/protein directed IgG production, as, for example anti-TT IgG, may be not. For this reason, sera obtained during the initial immunization trial with 100 *μ*g NP_21_-CG were additionally subjected to anti-CG IgG ELISA. As shown by Figure 4, CD18^−/−^ mice were well able to mount anticarrier/protein-specific IgG titers to a similar extent as they mounted antihapten-specific IgG titers if provided with a suitable antigenic stimulus. Our studies demonstrate that reduced anti-NP IgG levels (Figure 4(a)) were paralleled by a decrease in anti-CG IgG levels (Figure 4(b)), both in WT and in CD18^−/−^ mice and preclude a general deficiency of producing protein-specific antibody in our murine LAD1 model.

However, in contrast to WT, CD18^−/−^ mice exhibited a definite impairment in their primary and secondary IgG responses upon decreased antigen quantity or hapten coupling. Whereas upon doses as low as 10 *μ*g NP_21_-CG, CD18^−/−^ mice revealed a significant reduction of NP- and CG-specific IgG production only during the secondary immune response (*P* < 0.05), a reduced NP/CG ratio of 4/1 (*P* < 0.05), and more distinctly, uncoupled CG (*P* < 0.005) significantly lowered antigen-specific IgG throughout all time points analyzed. When the hapten/protein ratio was decreased to 4/1, the gap between the CD18^−/−^ and WT cohort was more pronounced than after a mere reduction of the antigen dose to 10 *μ*g NP_21_-CG. Furthermore, even after injection of a dose as high as 100 *μ*g nonhaptenated CG, CD18^−/−^ mice mounted only very poor anti-CG IgG titers that remained more than 4 logs below the WT cohort, also after boosting (Figure 4(b)). In the CD18^−/−^ cohort that had been administered 100 *μ*g NP_4_-CG, anti-CG IgG levels still were 3 logs below those of the WT cohort. Using 100 *μ*g NP_21_-CG, CD18^−/−^ mice produced anti-CG IgG at WT levels. Altogether these data demonstrate a gradual dependence of adaptive humoral immunity in CD18^−/−^ mice on CG haptenation. These data furthermore indicate that the deficiency in adaptive humoral immunity of CD18^−/−^ mice can be compensated by the amount of antigen injected for immunization. However, haptenation of proteins is pivotal and can effectively rescue antigen-specific IgG production in CD18^−/−^ mice turning a weak protein antigen into a strong immunogen in this system.

### 3.5. CD18^−/−^ Mice Secrete Normal Levels of Anti-TT IgG after Immunization with NP-TT

**Figure 5:**
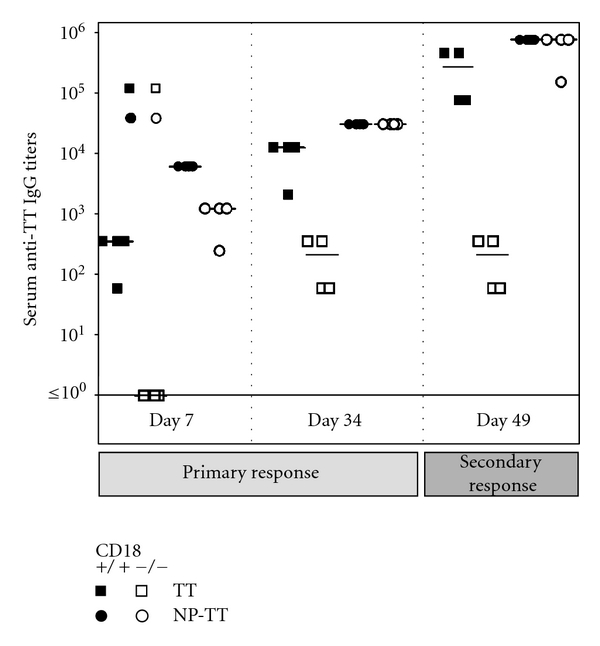
Adaptive immunity is functional upon immunization with NP-TT in CD18^−/−^ mice. Eight- to twelve-week-old CD18^−/−^ (open symbols) and WT (filled symbols) mice were immunized intraperitoneally with 2.0 Lf TT/alum (squares) or 2.0 Lf NP-TT/alum. For measurements of anti-TT IgG_1_, blood was obtained by bleeding from tail veins. Prior to analysis, sera were diluted 1 : 10, and then plated out on TT-coated plates in 1 : 5 dilution steps. Serum titers of anti-TT-specific IgG_1_ were determined from the last dilution step where the optical density was still above the background level of the assay. For assessment of the primary immune response, results from sera collected at days 7 and 34 are displayed. Besides, measurements for day 49 are shown, and depict IgG_1_ titers representative also for further time points assessed during secondary immune responses. Bars represent the median of each group.

To test the validity of our conclusions drawn from immunization with the haptenated carrier NP-CG also for other T-dependent antigens, TT vaccine was conjugated with NP, and subsequently used for immunization as described above.  Figure 5 shows a slight initial delay of anti-TT IgG production at day 7 after first injection of 2 Lf NP-TT, reflecting results obtained during the primary immune response to 100 *μ*g NP_21_-CG. At all later time points assessed, production of anti-TT IgG in CD18^−/−^ mice occurred at equal levels as in WT mice, compared to immunization with 2 Lf nonconjugated TT, which had failed to induce sufficiently high anti-TT IgG titers. Collectively, these findings demonstrate, for the first time, that the elicitation of a full adaptive immune response can be obtained upon immunization with a highly haptenated TT analogue in complete absence of CD18. This argues towards the use of highly haptenated antigens as vaccines in LAD1.

## 4. Discussion

Effective induction of an adaptive immune response relies on a fine-tuned orchestration of cell-cell interactions. This requires integrity of functional as well as structural components of the immune response. Despite the involvement of CD18 in cognate interactions between APCs, T and B lymphocytes, and a marked impairment of the adaptive immune response in patients deficient in CD18 (LAD1) [23, 24, 35], we here report that a suitable, multivalent antigenic stimulus can distinctly overcome the necessity of CD18 for adhesion and intracellular signaling leading to a robust antigen-specific humoral immune response* in vivo*. Using the protein antigens CG or TT gradually haptenated with NP as immunizing agents, we could induce a full adaptive immune response in a murine model of LAD1. This shows that CD18 is dispensable for the elicitation of a complete adaptive immune response *in vivo* under the here further defined vaccination conditions. We here demonstrate that CD18 deficiency with specific defects in intercellular adhesion required for cellular communication and activation at several stages of adaptive immunity can nevertheless be overcome in CD18^−/−^ mice *in vivo* by increasing antigen concentrations or by modification of antigen quality towards carrier haptenation. These results are potentially valuable for patients suffering from LAD1 or similar immunodeficiency.

A critical event in the initiation of adaptive immune responses is the activation of T lymphocytes. LFA-1 (CD11a/CD18) is known to participate critically in the biochemical and structural organization of immunological synapses during T-cell activation [14]. It is a prerequisite for a sustained TCR/MHC-peptide engagement [1, 3, 40] to achieve commitment to cytokine synthesis (IL-2, IFN*γ*) [9] and T-cell proliferation [7, 8]. Accordingly, our previous *in vitro* data showed that CD18^−/−^ mice exhibited a severely impaired activation of T-cells in MLR [25]. However, full T cell activation was possible, when priming was done using a sufficient amount of antigen, antigenic restimulation [36], or IL-2 substitution [36, 41].

We here show that the requirement for LFA-1 also in T : B cell contacts is overall not essential for generating an adaptive immune response* in vivo*. Our major finding is that highly haptenated antigens do not depend on CD18 to elicit complete adaptive immune responses. This may be due to the fact that crosslinking of BCR does not require CD18. Indeed, increased numbers of epitopic binding sites obtained by multivalent haptenation cause a profound reduction in both the minimal concentration and affinity requisites for B-cell activation [20, 42]. One key mechanism likely to contribute to this phenomenon may be the marked increase in IL-2 release due to an efficient crosslinking of BCRs by multivalently haptenated antigen [43, 44]. This may have contributed to the rescue of the previously demonstrated deficiency in IL-2 release in absence of LFA-1 or CD18 [9, 36, 41]. Accordingly, an enhanced release of IL-2 from B cells due to crosslinking by polyhaptenated antigen may have compensated for the intrinsic defect of CD18^−/−^ T cells to secrete IL-2. In addition, B-APC may be superior to DC in antigen presentation and subsequent activation of CD4^+^ T cells upon encounter with protein antigen as compared to peptide antigen [45]. Haptenated carriers even multiply this effect. Altogether, these mechanisms may contribute to the herein observed effect that polyhaptenated proteins help to mount an effective immune response even under conditions of an impaired synapse formation in the absence of CD18.

Our results furthermore provide circumstantial evidence that several redundant pathways may exist *in vivo* substituting for each other to obtain a sufficient immune response. However, impairment of a distinct accessory pathway may limit or suppress immune responses that were previously robust by critically elevating activation thresholds, as has been shown upon injection of TT in CD18^−/−^ mice. Such thresholds have earlier been described in detail for the different types of cellular interactions *in vitro *[1, 2, 5, 14, 36]. *In vivo*, also spatial availability of both lymphocytes and APCs in a timely highly regulated fashion is pivotal for the elicitation of adaptive immunity [46]. But with regard to interstitial tissue locomotion of leukocytes (i.e., in three-dimensional environments), the role of CD18 (and also of other integrins) seems to be rather limited [6, 47]. Nevertheless, in CD18^−/−^ mice, structural integrity of lymphoid organs is severely affected [25]. This is most likely due to impairments in cell trafficking in the context of systemic leukocyte recirculation [27, 48, 49] and the overall proinflammatory situation [28] in these mice.

Our data of an intact class switch and memory function support former reports. These reports revealed that a structural integrity of GC, which have been commonly called to account for class switch, affinity maturation, and memory B-cell generation [50, 51], is not compellingly required to mount a full adaptive immune response [51–54]. Also, mice deficient in Lyn kinase (LynK) exhibit absence of GC combined with a widely functional humoral immune response showing functional antibody production, class switch, or even affinity maturation [55]. Interestingly, both CD18 and LynK, apart from costimulatory signaling, are involved in cell-cell adhesion stabilizing membrane contacts required for cognate synapses in GC [56].

Several reports reveal that antigen-driven clonal selection of antibody-forming cells (AFCs) leading to an effective affinity maturation of secreted Ig strictly requires Th : B cell cooperation but can take place independently of classical GC structures [32, 37, 52], even in compartments such as the BM [33, 57, 58]. However, B-cells with GC phenotypic markers seem to be inevitable as an intermediate step for AFC generation, independent of the respective type of lymphoid tissue where the AFC emerge [32, 58]. B cell maturation to AFC can be even achieved *in vitro* without forming the typical complex GC structures but still traversing intermediate stages with expression of GC markers [59]. GC phenotypic cells with specificity for the injected antigen were also detected in peripheral lymphoid tissues of CD18^−/−^ mice at low numbers, although classical GC with an analogous specificity remained absent. Besides, careful examination revealed no hints for a compensatory hosting of GC structures or cells in the BM of CD18^−/−^ mice. We therefore conclude that disseminated GC-phenotypic cells were functional in mediating an adaptive immune response including a normal affinity maturation of antibody in CD18^−/−^ mice.

In summary, our data suggest that functional adaptive immunity in murine LAD1 depends on specific properties of the employed immunogen. Demonstrating that absence of CD18 largely reduces the spectrum of suitable immunogens, our data provide further insight into the role of *β*_2_ integrins in adaptive immunity yielding novel results with regard to the complex *in vivo* situation. Based on our data, we suggest that distinct features of an immunization with highly haptenated NP conjugates only could lead to a rescue of function, though not to a rescue of structure with immunogen-specific GC remaining absent. Our data may stimulate further investigations in LAD1 patients.
